# Trophic position determines the persistence of neotropical understory birds after forest disturbance

**DOI:** 10.1002/ece3.11370

**Published:** 2024-05-06

**Authors:** Bernhard Paces, Wolfgang Wanek, Christian C. Voigt, Saskia Schirmer, Paulus Leidinger, Christian H. Schulze

**Affiliations:** ^1^ Department of Botany and Biodiversity Research University of Vienna Vienna Austria; ^2^ Department of Microbiology and Ecosystem Science, Center for Microbiology and Environmental Systems Science University of Vienna Vienna Austria; ^3^ Department of Evolutionary Ecology Leibniz Institute for Zoo and Wildlife Research Berlin Germany; ^4^ Zoological Institute and Museum University of Greifswald Greifswald Germany

**Keywords:** forest disturbance, secondary forest, stable isotope analysis, trophic niche width, trophic position, understory bird species

## Abstract

Habitat loss and degradation are key drivers of the current biodiversity crisis. Most research focuses on the question of which traits allow species to persist in degraded habitats. We asked whether a species' trophic position or niche width influences the resilience of species in degraded habitats and to what extent habitat degradation affects trophic interactions between species. We used nitrogen isotope ratios (^15^N:^14^N, expressed as δ^15^N value) to quantify and compare trophic positions and niche widths of understory birds inhabiting old‐growth and young secondary forests in the Pacific lowlands of Costa Rica. We found that a species' trophic position rather than its trophic niche width determined its persistence in secondary forests. Species feeding at lower trophic levels in old‐growth forests were less likely to persist in secondary forests than those occupying a higher trophic position in old‐growth forests. This pattern is likely induced by the occurrence of relatively large‐bodied habitat specialists with a flexible and high‐trophic level diet in secondary forests. These habitat specialists likely caused generalist bird species to lower their trophic position relative to conspecifics in old‐growth forests. Regarding trophic niche widths, species in secondary forests tend to have larger niche widths than old‐growth forest species. However, as old‐growth forest specialists and generalists did not differ in their niche widths, no systematic effect of trophic niche width on species persistence after forest disturbance was found. This is the first study that shows a systematic effect of trophic position on the persistence of a wide range of bird species in a disturbed forest ecosystem. It therefore provides important insights into species' responses to habitat degradation and the conservation value of secondary forests. To improve habitat quality for old‐growth forest birds and facilitate avian seed dispersal, the creation of large contiguous forest patches should be prioritised when implementing reforestation measures.

## Graphical abstract

We studied whether an understory bird species' trophic position or niche width determines its persistence after forest disturbance in the Pacific lowlands of Costa Rica. Using stable isotope methodology, we found a significant effect of trophic position: Species feeding at low trophic levels in old‐growth forests were less likely to persist in secondary forests. The dataset provides insights into species' responses to habitat degradation and the conservation value of the secondary forests in the study area, which are useful for planning of future conservation measures in the region.

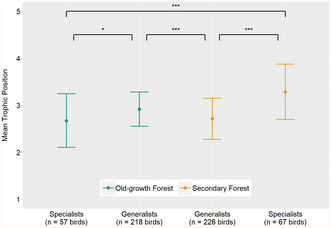

## 1 INTRODUCTION

Habitat loss and degradation are main drivers of species extinction in the current biodiversity crisis (Barnosky et al., 2011). In tropical biodiversity hotspots such as Mesoamerica (Brooks et al., 2002), biodiversity appears to be even more vulnerable to habitat degradation than in temperate regions (Edwards et al., 2014; Stratford & Robinson, 2005). While tropical rainforest ecosystems are highly threatened by ongoing deforestation (Keenan et al., 2015; Potapov et al., 2017), secondary forest areas are expanding and therefore play an important role in the conservation of tropical forest biodiversity (Barlow et al., 2007; Chazdon et al., 2009; Wright, 2005). Thus, research is increasingly focusing on the question of what traits allow some species to use degraded habitats while others do not (Koh et al., 2004; Newbold et al., 2013; Öckinger et al., 2010; Pavlacky et al., 2015; Socolar & Wilcove, 2019). A general understanding of the factors involved is not only vital for determining the response of a species to habitat degradation and the resulting conservation implications but also enables assessments of potential consequences for the functioning of whole ecosystems and the provisioning of ecosystem services (Hooper et al., 2005; Newbold et al., 2013; Sekercioğlu et al., 2004).

In particular, little is known about how a species' trophic position or trophic niche width influences its potential to persist in degraded habitats and about the way trophic interactions change due to forest degradation (Edwards et al., 2013; Hamer et al., 2015), although the consequences of changes in the network of trophic interactions can be far‐reaching (Estes et al., 2011). For instance, both habitat degradation and trophic changes in an ecosystem are among the causes leading to the observed increase in infectious diseases all around the globe (Pongsiri et al., 2009; Smith et al., 2014; Whitmee et al., 2015).

For birds, which are the best‐studied animal taxon in tropical regions (Hill & Hamer, 2004), it is well documented that forest habitat specialists are more sensitive to forest disturbance than habitat generalists (Newbold et al., 2013; Owens & Bennett, 2000). This pattern was found to be linked – among other factors – to dietary specialisation and trophic niche width (Edwards et al., 2013; Newbold et al., 2013). Edwards et al. (2013) showed that species only occurring in unlogged forest (habitat specialists) had a narrower trophic niche width than habitat generalists occurring in unlogged and selectively logged forest on the island of Borneo. Thus, a bird species' trophic niche width determined its persistence after forest disturbance when analysing presence–absence data for a wide array of understory bird species (Edwards et al., 2013). However, in the same study area changes in the abundances of insectivorous birds post‐logging were related to the trophic position of these species. Small understory species with lower trophic positions were less adversely affected by forest disturbance than large ground‐feeding species occupying higher trophic positions (Hamer et al., 2015). These contrasting results led to a call for additional data on this highly relevant conservation issue (Hamer et al., 2015). Here we study, for the first time in the Neotropics, how disturbance affects the trophic position and niche width of forest understory birds in forests exposed to different disturbance regimes. While Edwards et al. (2013) and Hamer et al. (2015) analysed the effects of selective logging on the trophic position and trophic niche width of understory bird species assemblages, we compared old‐growth forest and young secondary forest. This difference was deliberately chosen, not only to better understand the conservation value of this globally emerging forest type (Wright, 2005), but also because bird species assemblages may respond differently to different forms of forest disturbance (Durães et al., 2013; Moura et al., 2013).

The trophic position of an organism represents the number of trophic links separating it from the producer level (Thompson et al., 2007) and can be quantified using stable isotope analysis (Bearhop et al., 2004; Layman et al., 2012; Post, 2002). The ratio of ^15^N to ^14^N (expressed as the δ^15^N value) in the tissue of an organism is enriched by ~2‰–3‰ in relation to the food source, which eventually leads to gradual increases of δ^15^N values along the food chain (Caut et al., 2009; Perkins et al., 2014; Vanderklift & Ponsard, 2003). Therefore, δ^15^N values indicate the organism's trophic position during the period of synthesis of the respective tissue (Bearhop et al., 2003; Blüthgen et al., 2003; Caut et al., 2009). Additionally, the variation in the trophic position of individual birds from the same species can provide a measure for the trophic niche width of a specific population (Bearhop et al., 2004).

In this study, we used stable isotope analysis to compare the trophic positions and trophic niche widths of understory bird species between old‐growth forests and secondary forests in the Pacific lowlands of southwestern Costa Rica, aiming to test the following three hypotheses:

1. The trophic positions of birds in secondary forests are higher than in old‐growth forests due to dietary shifts and increased trophic positions of prey species (Blüthgen et al., 2003; Edwards et al., 2013; Hamer et al., 2015; Kemp, 2018; Woodcock et al., 2013).
2. The trophic niche widths of old‐growth forest bird species are greater than those of secondary forest species because individuals of species have access to more diverse food sources in old‐growth forests (Edwards et al., 2013; Kemp, 2018).
3. A species' trophic niche width rather than its trophic position determines which of the old‐growth forest species can persist in the secondary forests, with dietary specialists being more vulnerable to habitat destruction than generalists (Edwards et al., 2013).

## 2 METHODS

### 2.1 Study area

The study area was located in southwestern Costa Rica, east of the Golfo Dulce, on the edge of the Piedras Blancas National Park and the Esquinas Rainforest near the village La Gamba and the Tropical Research Station La Gamba (N 08°42.063′, W 083°12.102′, 70 m a.s.l.). This area is considered a biodiversity hotspot for both fauna and flora (Weissenhofer, Huber, et al., 2008), with more than 300 recorded bird species (Tebb, 2008). These include several range‐restricted species that qualify the region as an Endemic Bird Area (EBA 021: South Central American Pacific Slope). More than 50% of the EBA's range‐restricted bird species are abundant in the forests around the Golfo Dulce (BirdLife International, 2023; Stattersfield et al., 1998). These forests are classified as tropical lowland wet forests and form the most common natural vegetation type in the study area (Weissenhofer, Huber, et al., 2008).

The climate of the region is characterised by high annual precipitation (~6000 mm), with most rainfall from August to November (~700 mm per month) and January to March being the driest months (~200 mm per month; Weissenhofer & Huber, 2008). The mean annual temperature is 28.5°C and is relatively constant throughout the year (Weissenhofer & Huber, 2008).

### 2.2 Selection of mist netting sites

**FIGURE 1:**
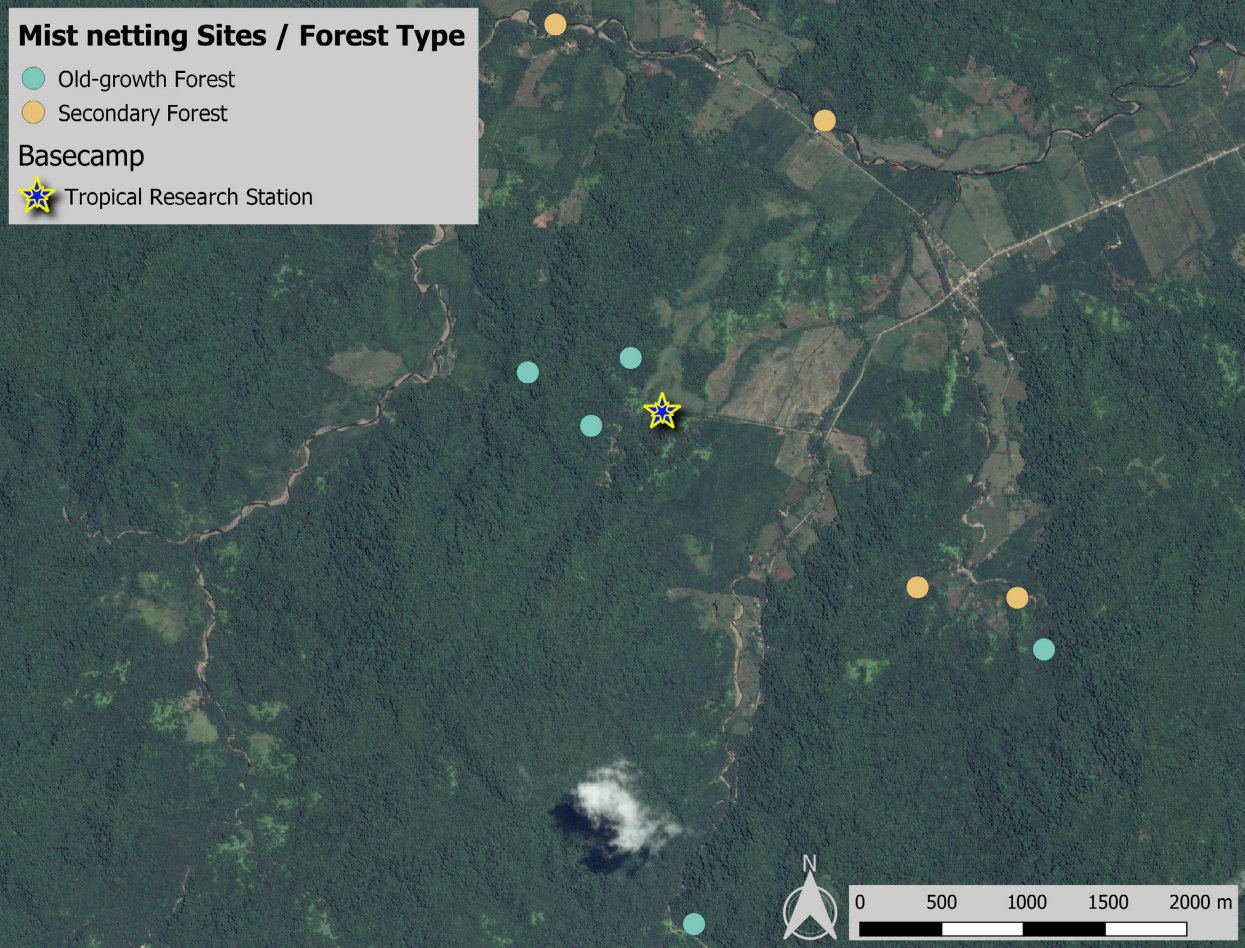
Map indicating the locations of the nine sampling sites and the respective forest type surrounding them. The location of the Tropical Research Station La Gamba is provided for orientational reasons only. Source of satellite image: Google © 2023 CNES/Airbus, Maxar Technologies.

We conducted this study at five old‐growth and four young secondary forest sites (Figure 1). We chose the respective spatial replicates of the two forest types according to the existing vegetation maps of the region (Höbinger et al., 2012; Weissenhofer, Huber, et al., 2008) and a map identifying plots of land where reforestation measures had been carried out in the past 15 years (Weissenhofer, Barquero, et al., 2008). The secondary forest sites were characterised by dense undergrowth with some old remnant trees, which served as shadow trees on these pieces of land formerly used as pasture or farmland (Weissenhofer, Barquero, et al., 2008). They were located close to existing, much older forest fragments and were therefore selected for implementing reforestation measures, to close forest gaps and to serve as biological corridors (Weissenhofer, Barquero, et al., 2008). Studies in tropical forests have shown that mist netting sites separated by more than 200 m are statistically independent (Hill & Hamer, 2004; Whitman et al., 1998). In this study, the minimum distance between two sampling sites was 357 m (Figure 1) and, indeed, we did not recapture a single bird at two different sites during the entire study period.

### 2.3 Avifaunal and vegetation sampling

We conducted fieldwork between 16 November 2017 and 28 January 2018. At every site, we set up six mist nets (Ecotone, Poland; length: 12 m, height: 2.5 m, number of shelves: 4, mesh size: 2 nets with 16 mm, 2 nets with 30 mm, 2 nets with 45 mm) along a linear transect to trap understory birds. Mist‐netting was conducted at a given site from 05:30 AM until 03:00 PM on the first sampling day and from 05:30 AM until 11:00 AM on the second day. If heavy rain forced us to close the mist nets, the missing trapping time was added to the end of the period whenever possible. After each two‐day period, we moved the nets and set them up at the next site for the following two consecutive sampling days. We conducted four sampling rounds. Within those, we randomly chose the order of the sites, but we did not sample a site twice within < 8 days. In the last sampling round, we added three mist nets (Ecotone, Poland; length: 12 m long, height: 2.5 m, number of shelves: 4, and mesh size: 16 mm) at every transect to increase the sample size. However, this was not possible at two secondary forest sites due to unsuitable terrain. Nevertheless, we obtained a relatively balanced sample for data analysis (see: Section 3).

We identified trapped birds to species level following Garrigues and Dean (2014) and Stiles and Skutch (1989). The nomenclature used in this article follows Garrigues and Dean (2014). English names are used throughout the text, and a table with corresponding scientific names is provided in Table A1. To avoid pseudoreplication, we marked trapped birds individually using plastic colour rings or metal bird rings. From each bird, we took nail clippings of its central front and rear claws and stored them in labelled vials for stable isotope analysis as claw material integrates the bird's diet over a period of several weeks (Bearhop et al., 2003). We excluded migratory bird species and hummingbirds (Trochilidae) from nail clipping collection because claw material of the former almost certainly would not have grown entirely in the sampling area (Bearhop et al., 2003) and because of the high risk of injury to the small feet of the latter. Standard morphological measurements were taken prior to release following Eck et al. (2011), such as body mass and tarsus length to facilitate the interpretation of the main results of this study.

In addition, we collected leaves along the mist nets halfway through the sampling period to determine the baseline δ^15^N signatures of the primary producers at every site, against which the δ^15^N values of the collected claw material could be assessed (Woodcock et al., 2012). Every 3 m we picked a leaf, either from 0.1 m, 1 m or 2 m above the ground. Thus, we collected 24 leaves (8 per height class) per site, placed them in a drying oven at 40–50°C for 24 h and then stored them in labelled paper bags. Altogether, we collected 9 leaf samples, one at each study site in old‐growth forest (*n* = 5) and one at each study site in secondary forest (*n* = 4).

Moreover, to facilitate data interpretation, we also collected fruits, moths and spiders at every transect halfway through the study period, which again resulted in nine samples per resource type (5 from old‐growth forests and 4 from secondary forests). All these samples were dried and stored in the same way as the leaves. Fruits, moths and spiders represent important food items for birds and different trophic positions in the local food webs. Moths (herbivores: trophic level two) were caught with a light‐trap, which we attached to a tree in the middle of the transect and operated for one night. We collected caught animals in the early morning and put them in a freezer for several hours before drying them. To reduce the heterogeneity of moth samples, only individuals of groups most likely feeding on vascular plants were considered (e.g. Lithosiinae which often feed on lichens were excluded; compare Adams et al., 2016). Fruits and spiders were collected by hand around the mist netting transects. We picked all apparently different kinds of fruits we found in a radius of 50 m around the transects. We cut large fruits into pieces before drying and aimed to include different states of ripening in our sample. Thus, if we found a fruit type in different colourations or states of consistency, we picked one representative fruit for each colouration or state. We also collected 10 morphologically different specimens of spiders (carnivores: trophic level three) within a radius of 50 m around the mist nets and handled the sample in the same way as the moth samples. Finally, we had one composite fruit, moth and spider sample for every transect.

### 2.4 Stable isotope analyses

All stable isotope analyses were performed at the Stable Isotope Laboratory of the Leibniz Institute for Zoo and Wildlife Research (IZW), Berlin, Germany. We only analysed claw samples from bird species with at least *n* ≥ 5 replicates in one forest type. Prior to analysis, we treated all samples from animals with a 2:1 chloroform/methanol solution (v/v) for 24 h to remove lipids and external contaminants. For larger moths, we selected the thorax for isotopic analysis, assuming that this body part does not deviate significantly from others with respect to isotopic composition. Then, we re‐dried all samples for 24 h in a drying oven at 50°C and ground leaves, fruits, moths, spiders and claws in a ball mill. Hereafter, we used a high‐precision balance to transfer 0.5 mg of animal samples and 1.5 mg of plant samples into tin capsules. We used an elemental analyser (Flash EA 1112; Thermo Fisher Scientific, Bremen, Germany) connected via a ConFlo III interface to a Delta V Advantage isotope ratio mass spectrometer (all Thermo Scientific, Bremen, Germany). We report values in the δ^15^N notation as parts per mil (‰) deviation of the ratio ^15^N:^14^N of atmospheric nitrogen. The precision of the measurements was always better than 0.15‰ for laboratory standards.

We calculated the trophic position of each sampled understory bird as *λ* + (δ^15^N_bird_ – δ^15^N_baseline organism_) / *E*, where *λ* is the trophic level of the organisms used to estimate δ^15^N_baseline organism_ (*λ* = 1 for plants used in this study) and *E* is the enrichment in δ^15^N per trophic level (Post et al., 2000). For the value of δ^15^N_baseline organism_, we used the δ^15^N signature of the 24 leaves collected from the same mist netting site from where the bird's claw material was collected (Woodcock et al., 2012). According to two extensive reviews, we chose a value of *E* = 2.5 as an appropriate trophic enrichment factor for birds (Caut et al., 2009; Edwards et al., 2013; Vanderklift & Ponsard, 2003). We calculated the trophic niche widths for each species as the standard deviation of the sampled individuals' mean trophic position. We used the same equation and the δ^15^N values from the fruit, moth and spider samples to calculate the respective trophic position of these samples. All calculations of trophic positions were based on the raw δ^15^N values of the samples.

### 2.5 Statistical analyses

Statistical analyses were conducted to test for differences in trophic position and niche width between four occurrence types of the sampled bird species (‘old‐growth forest specialists’, ‘old‐growth forest generalists’, ‘secondary forest generalists’ and ‘secondary forest specialists’). Therefore, we assigned each species (and their respective claw samples) to one occurrence type based on its presence in our samples from old‐growth forests, secondary forests or both forest types. A species was only classified as characteristic of one of the forest types if it occurred at more than one study site of that specific forest type, because records at a single site might occur by chance. As we only analysed claw samples from species with *n* ≥ 5 samples in one forest type, species with smaller sample sizes were neither assigned to an occurrence type nor included in any statistical analyses.

We used a phylogenetically informed Gaussian Bayesian Markov chain Monte Carlo generalised linear mixed model (MCMCglmm) in R v. 3.5.1 (package: MCMCglmm v. 2.29) with a fixed effect for occurrence type and a random effect for phylogenetic information to test for differences in the trophic positions of individual birds between the four occurrence types (Hadfield, 2010, 2019; Hadfield & Nakagawa, 2010; R Core Team, 2018). To account for species affiliation and phylogenetic non‐independence between species in this model, we used edge lengths of phylogenetic consensus trees as random effects. Therefore, we downloaded two phylogenetic tree samples, each consisting of 1000 phylogenetic trees, from the BirdTree project (http://birdtree.org; Jetz et al., 2012), one using the backbone by Hackett et al. (2008) and one using the backbone by Ericson et al. (2006). We generated two 50% consensus trees based on these samples using the least‐squares method in the R package phytools v. 0.6‐99 (Revell, 2012). Consequently, we ran the MCMCglmm twice, with the species‐specific edge lengths of the two different consensus trees included as random effects. As the results differed only marginally, we calculated all reported outputs using the backbone by Hackett et al. (2008). We used weakly informative priors (*V* = 1, nu = 0.002) corresponding to an inverse‐Gamma distribution and specified 5,000,000 Monte Carlo iterations with a burn‐in of 1000 and thinning of 500. The models converged according to visual inspection of their trace plots, and the Gelman‐Rubin statistic was always < 1.1 for all parameters (Gelman et al., 2014; Roy, 2020). We used the parameter estimations from this trophic position model (TPM) to calculate the mean trophic position for every occurrence type and its 95% credible intervals (Hadfield, 2010; Hadfield & Nakagawa, 2010).

To test for differences in species' trophic niche widths between the four occurrence types, we also used a MCMCglmm in R v. 3.5.1 (Hadfield, 2010, 2019; Hadfield & Nakagawa, 2010; R Core Team, 2018). However, we did not include edge lengths of a phylogenetic tree as random effect in this model, because our sample size was too small and model convergence was otherwise not achieved. Instead, to check for phylogenetic non‐independence, we calculated pairwise Mantel tests with 9999 permutations in Past v. 4.02 between the phylogenetic relatedness matrices of the set of species in each forest type and their respective Euclidean distances in trophic niche widths (Hammer et al., 2001). We calculated every pairwise comparison twice, once with the phylogeny based on the backbone by Hackett et al. (2008) and once with the one based on the backbone by Ericson et al. (2006; 4 Mantel tests, all *p* > .5). The model specifications of this trophic niche width model (TNWM) were the same as for the TPM. The model converged according to visual inspection of its trace plot, and the Gelman‐Rubin statistic was < 1.1 for all parameters (Gelman et al., 2014; Roy, 2020). We used the parameter estimations from TNWM to calculate the mean trophic niche width for every occurrence type and its 95% credible intervals (Hadfield, 2010; Hadfield & Nakagawa, 2010).

To check for spatial autocorrelation in this dataset, we calculated a linear model of the species' mean trophic positions at every sampling site within each forest type in R v. 3.5.1 (R Core Team, 2018). We used the site‐specific model residuals hereafter to calculate a matrix with Euclidean distances between those, and checked for a significant correlation with a matrix containing all pairwise linear distances between the sites using a Mantel test with 9999 permutations in Past v. 4.02 (*p* = .85; Hammer et al., 2001). We repeated this procedure three times, testing the site‐specific residuals of the mean trophic positions of the three most caught bird species for a significant correlation with the pairwise linear distance matrix (all *p* > .3). Therefore, we are confident that the spatial arrangement of our study sites did not affect our analyses.

To facilitate interpretation of the obtained results from the stable isotope data analyses described above, we conducted three additional analyses, which were not directly connected to the research hypotheses, but nevertheless relevant for the discussion.

1. We tested for differences in the trophic positions of the collected fruit, moth and spider samples between forest types using Mann‐Whitney*‐U*‐Tests calculated in Past v. 4.02 (Hammer et al., 2001).
2. We tested for differences in species' mean body mass between habitat specialists from old‐growth forests and secondary forests using a Mann‐Whitney*‐U*‐Test calculated in Past v. 4.02 (Hammer et al., 2001).
3. We tested for differences in size‐corrected body mass between habitat generalists from old‐growth forests and secondary forests using a MCMCglmm in R v. 3.5.1 (Hadfield, 2010, 2019; Hadfield & Nakagawa, 2010; R Core Team, 2018) with a fixed effect for forest type and a random effect for phylogenetic information. Therefore, we used the edge lengths of a 50% consensus tree generated from 1000 phylogenetic trees based on the backbone by Hackett et al. (2008), as described in more detail for the TPM above. Model specifications were the same as for the TPM. The model converged according to visual inspection of its trace plot, and the Gelman‐Rubin statistic was < 1.1 for all parameters (Gelman et al., 2014; Roy, 2020). Size‐corrected body mass was calculated for every relevant bird as follows: (body mass / tarsus length) * (mean tarsus length of species in respective forest type).

## 3 RESULTS

Raw δ^15^N values of the leaf samples used to calculate trophic positions of the sampled birds ranged from −1.53 to −0.01 (mean = −0.726; SD = 0.648) in old‐growth forests and from −0.98 to 0.83 in secondary forests (mean = −0.03; SD = 0.795).

**FIGURE 2:**
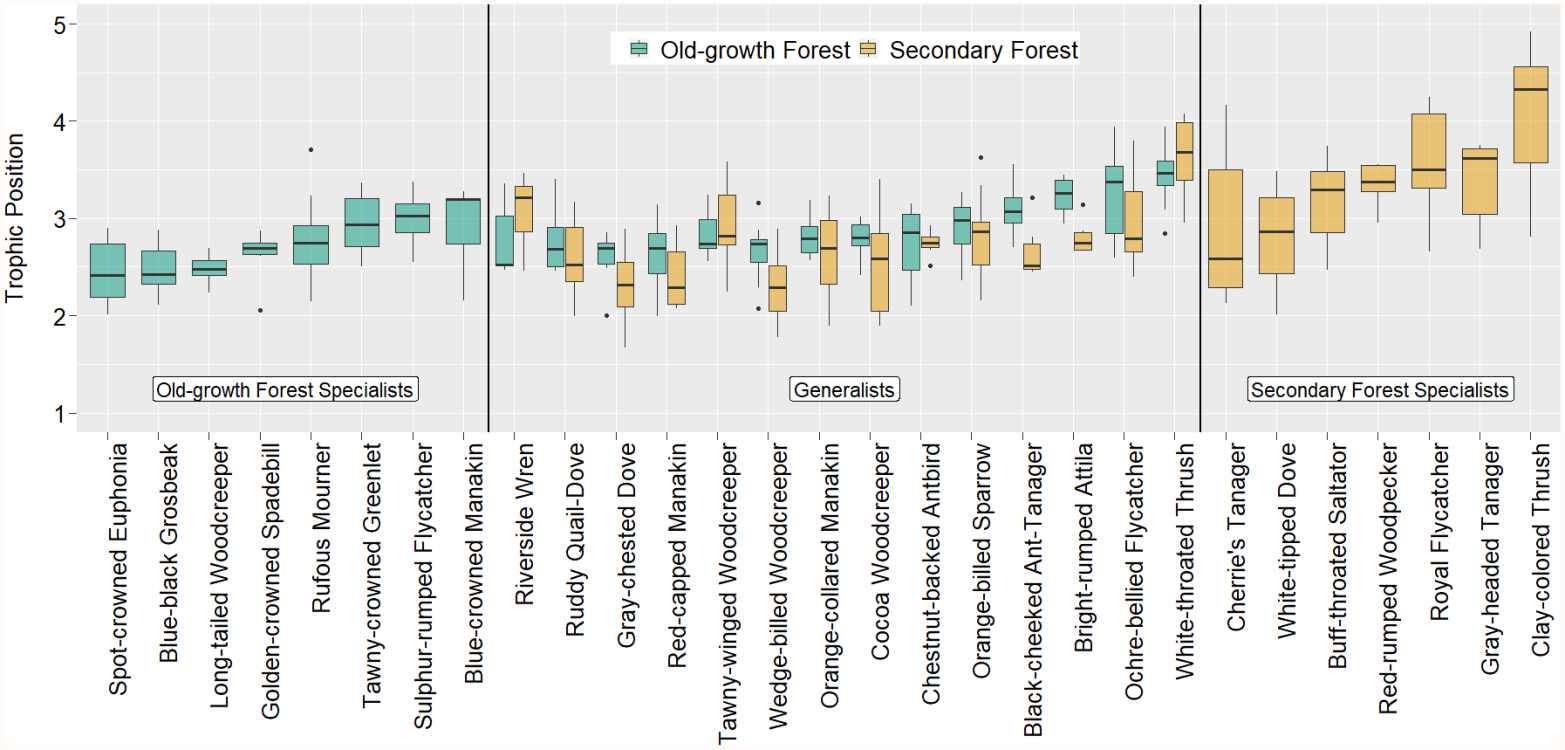
Calculated median trophic positions of all bird species analysed in this study. Boxes depict the interquartile range, whiskers minimum/maximum values within 1.5 times the interquartile range. Species occurrence types relevant for data interpretation are separated by continuous black vertical lines. Within these, species are ordered by increasing median trophic position in old‐growth forests or secondary forests, respectively. *n* ≥ 5 claw samples for every species in each forest type.

We obtained δ^15^N values from 22 bird species (*n* = 275 birds) in old‐growth forests (8 species categorised as habitat specialists: *n* = 57 birds, 14 species as habitat generalists: *n* = 218 birds) and from 21 species (*n* = 293 birds) in secondary forests (7 species categorised as habitat specialists: *n* = 67 birds, 14 species as habitat generalists: *n* = 226 birds; Figure 2).

**FIGURE 3:**
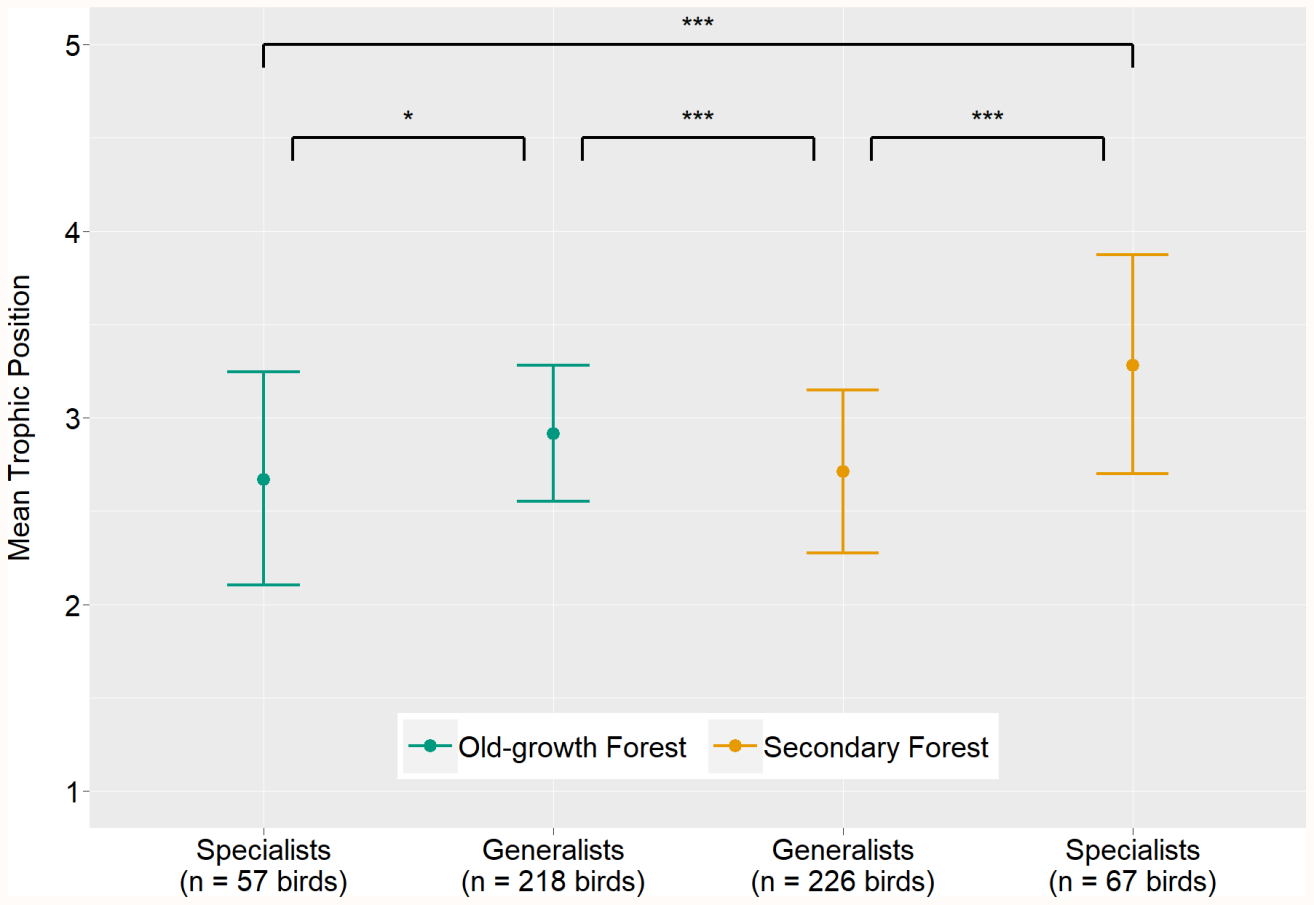
Posterior means (± 95% credible intervals) of the trophic positions of the four bird species occurrence types. Values were calculated using the trophic position model (TPM; Table A2). Indicated *p*‐values correspond to the respective pairwise species category comparisons (TPM; Table A2). *p < .05, ***p < .001.

Old‐growth forest specialists had a lower trophic position than old‐growth forest generalists (MCMCglmm, TPM: *p* = .017; Table A2; Figure 3). Therefore, trophic position determined species persistence in secondary forests. Old‐growth forest generalists significantly lowered their trophic position when they occurred in secondary forest (MCMCglmm, TPM: *p* < .001; Table A2). Compared to old‐growth forest generalists, secondary forest specialists had a higher trophic position (MCMCglmm, TPM: *p* = .002; Table A2; Figure 3). Consequently, secondary forest specialists also had a higher trophic position than secondary forest generalists and old‐growth forest specialists (MCMCglmm; Table A2; Figure 3).

**FIGURE 4:**
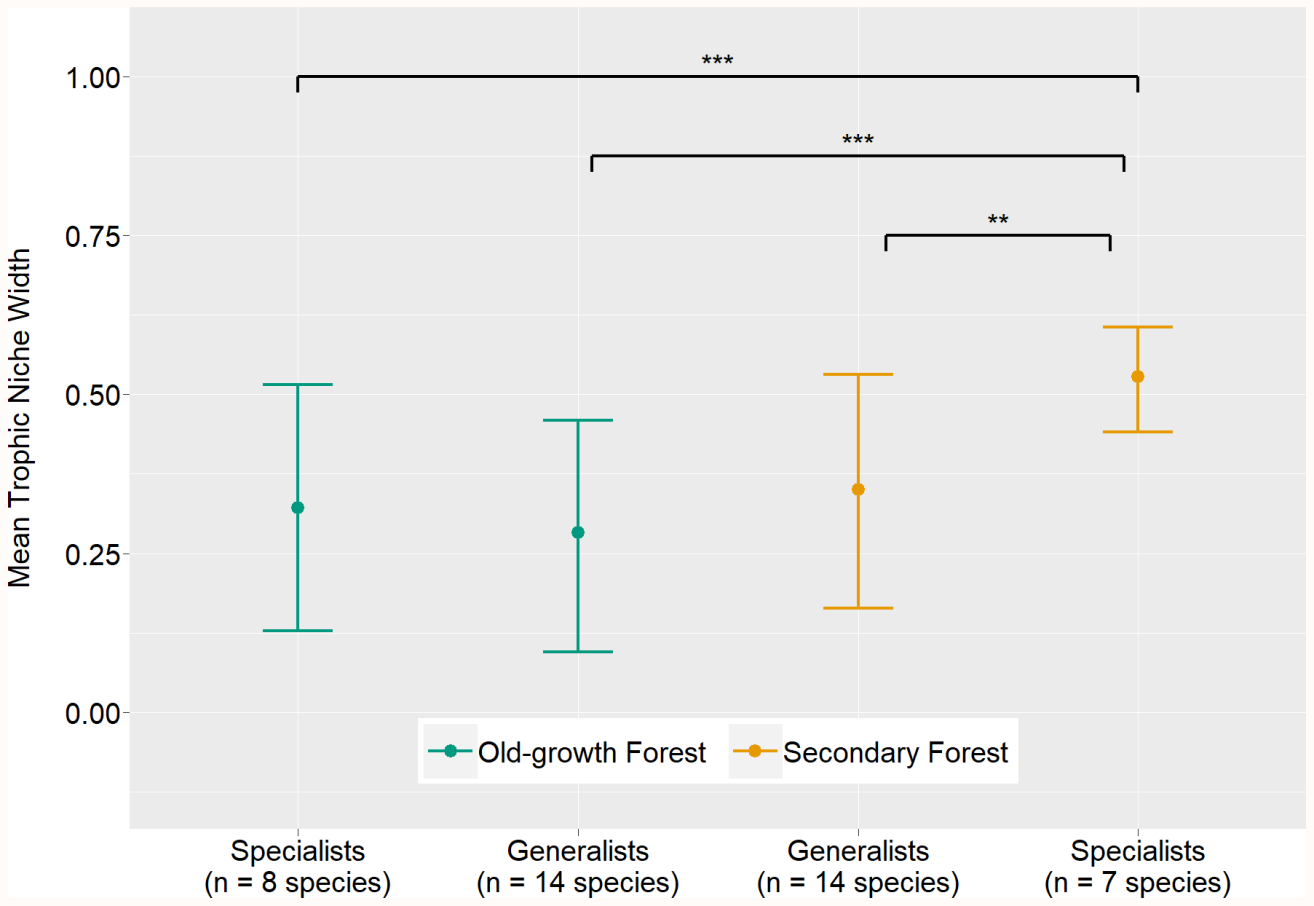
Posterior means (± 95% credible intervals) of the trophic niche widths of the four bird species occurrence types. Values were calculated using a trophic niche width model (TNWM; Table A3). Indicated *p*‐values correspond to the respective pairwise species category comparisons (TNWM; Table A3). **p < .01, ***p < .001.

Regarding trophic niche widths, secondary forest specialists differed significantly from all other groups (MCMCglmm, TNWM: all *p* ≤ .001; Table A3; Figure 4), while old‐growth forest specialists, old‐growth forest generalists and secondary forest generalists seem to be similar in their niche widths (Figure 4).

Additional analyses showed that

1. Trophic positions of the collected fruit, moth and spider samples around the study sites did not differ between forest types (3 Mann‐Whitney‐*U*‐Tests: *p* ≥ .27; Table A4).
2. Habitat specialists in old‐growth forests had significantly lower mean body mass than those in secondary forests (old‐growth forests: *n* = 8, median = 12.5 g; secondary forests: *n* = 7, median = 30.7 g; Mann‐Whitney‐*U* = 9, *p* = .032).
3. There was no difference in size‐corrected body mass between habitat generalists occurring either in old‐growth forests or in secondary forests, indicating that there is no substantial difference in body condition between forest types (MCMCglmm: *p* = .752; Table A5).

## 4 DISCUSSION

Mean trophic positions of species analysed in this study ranged from 2.3 to 4.1, which is generally in line with literature classifying the sampled species as frugivorous, omnivorous or insectivorous (Garrigues & Dean, 2014; Stiles & Skutch, 1989). Krebber (2019) analysed faecal samples of the understory bird species in the same study area and frequently found arthropod parts in the faeces of all studied bird species, even those regarded as obligate frugivores such as Manakin species (Pipridae). For Manakins, similar results were also found in eastern Ecuador (Fair et al., 2013). Therefore, mean trophic positions > 2 appear to be plausible even for frugivorous bird species such as Manakins (Edwards et al., 2013; Ferger et al., 2013). Insectivorous bird species are known to feed on fruits occasionally, especially in the rainforest understory. Therefore, trophic positions < 3 for presumed insectivores are frequently encountered in similar studies, as is a substantial overlap in static feeding guilds (Edwards et al., 2013; Ferger et al., 2013; Hamer et al., 2015; Herrera et al., 2003).

However, it should be noted that inaccuracies in the estimations of birds' trophic positions are an inherent issue in bulk stable isotope datasets (Martínez del Rio et al., 2009; Nielsen et al., 2018). Those can only be overcome by applying multiple methods of diet tracing to the same set of species (Nielsen et al., 2018), which was beyond the scope of this study. However, as species' diets and trophic niches are often difficult to assess, especially for small bird species occurring in dense rainforest understory, and as our resulting trophic positions are generally in line with the literature, we are confident that our approach is suitable to shed light on the outlined research hypotheses (Edwards et al., 2013; Hamer et al., 2015; Herrera et al., 2003; Layman et al., 2012).

Unexpectedly, we found lower trophic positions in secondary forest generalists compared to old‐growth forest generalists. Changes in trophic position do not necessarily indicate changes in the trophic position of prey species, but can also result from differences in the nitrogen isotopic signature of the baseline organisms used for the calculation of trophic positions or from varying body conditions between groups (Gorokhova, 2017; Hobson et al., 1993; Martínez del Rio et al., 2009; Woodcock et al., 2012). However, we accounted for local differences in baseline isotopic ratios by using sampling site‐specific δ^15^N values of plants in the calculation of trophic positions (Woodcock et al., 2012). In addition, there was no difference in size‐corrected body mass between generalists occurring either in old‐growth forests or in secondary forests, indicating no substantial difference in body condition between forest types. Therefore, we are confident that our findings represent fundamental changes in birds' trophic position after habitat degradation. This pattern is opposite to findings for birds, bats and ants from the island of Borneo, where species and/or individuals occurring in selectively‐logged rainforest had a higher trophic position than those in unlogged forest (Edwards et al., 2013; Hamer et al., 2015; Kemp, 2018; Woodcock et al., 2013). Blüthgen et al. (2003) also documented increased trophic positions of ants from naturally regenerating forest compared with mature wet forest in Australia, and this pattern is also known from the aquatic realm (Power et al., 2008). However, disturbance has also been shown to decrease trophic positions in ants and aquatic organisms (Fox et al., 2009; Gibb & Cunningham, 2011; Kim et al., 2019; McHugh et al., 2010).

In this study, we document for the first time a disturbance‐induced decrease in trophic positions of understory birds. A reduction in avian trophic position can be observed when birds feed on lower trophic levels and/or when their prey reduced its trophic position (Edwards et al., 2013; McHugh et al., 2010; Post et al., 2000; Post & Takimoto, 2007). Although the sample size was small, trophic positions of fruit, moth and spider samples did not differ between forest types. Thus, there is no reason to assume substantial changes in the trophic position of important avian dietary components. Interestingly, specialists in old‐growth forests had lower body mass than those in secondary forests, had a smaller trophic niche width than those in secondary forests and a lower trophic position than generalists occurring in old‐growth forests. However, the heavier secondary forest specialists had a higher trophic position than generalists in the same habitat and wider trophic niche widths. Therefore, it is more likely that the observed changes in trophic positions of habitat generalists are a result of competitively induced dietary shifts than a consequence of trophic changes in main food sources. However, this needs to be confirmed in future studies.

Following the definition of food chain length by Post and Takimoto (2007), we found a marked increase in food chain length from old‐growth forests (highest mean trophic position of all bird species: 3.46 ± 0.29) towards secondary forests (highest mean trophic position of all bird species: 4.09 ± 0.67). This is similar to findings by Hamer et al. (2015) and Woodcock et al. (2013) for birds and ants, respectively, who found an increase in food chain length from unlogged towards selectively logged forest on the island of Borneo. The reason for these observations could be the high productivity of certain disturbed rainforest types, such as selectively logged forest on the island of Borneo or secondary forests in the Pacific lowlands of Costa Rica (Berry et al., 2010; Hamer et al., 2015; Takimoto & Post, 2013; Wanek et al., 2008).

As we were only able to measure trophic niche width at the species level and not at the individual level, as was the case for trophic position, the sample size for comparing trophic niche widths between the four species occurrence types was small. Therefore, it is possible that additional sampling could provide even more insights into changes in trophic niche widths due to forest disturbance.

Because old‐growth forest specialists did not differ from old‐growth forest generalists in their trophic niche widths, we could not document a systematic effect of trophic niche width on species persistence after forest disturbance in our dataset. However, secondary forest specialists had larger trophic niche widths than secondary forest generalists and old‐growth forest specialists, thus indicating that habitat specialists are not necessarily dietary specialists. As dietary specialists are less likely to persist in disturbed habitats (Edwards et al., 2013; Newbold et al., 2013), larger trophic niche widths in secondary forest specialists would be expected. Interestingly, generalists also had slightly larger trophic niche widths when occurring in secondary forests, although this difference was not significant. Our data, therefore, do not support the hypothesis that trophic niche widths are compressed due to a restricted resource diversity at lower trophic levels in disturbed habitats, which is well documented for birds of more natural versus highly degraded neotropical landscapes (Navarro et al., 2021) but also for other taxa and ecosystems (Burdon et al., 2020; Crowley et al., 2012; Edwards et al., 2013; Kemp, 2018; Layman et al., 2007). This means that birds in our study consumed a similarly diverse diet in secondary forests compared to old‐growth forests, at least in terms of the trophic positions of their ingested food items (Edwards et al., 2013).

While data of the cited studies are – to our knowledge – most likely not affected by corridor effects, the secondary forest patches studied here were selected for reforestation measures because of their suitability to serve as biological corridors (Weissenhofer, Barquero, et al., 2008). As corridors increase the movement of organisms between habitat patches (Gilbert‐Norton et al., 2010), it is likely that mobile organisms using these temporarily increase the available resource diversity for birds (Resasco et al., 2012). Additionally, the catchment area around the mist nets from where we sampled birds may also be increased due to corridor effects.

Competition is another factor which can influence dietary breadth, with a higher amount of competitors leading to a narrower dietary or trophic niche (Fründ et al., 2013; Inouye, 1978; Kim et al., 2019; Pacala & Roughgarden, 1982). In our dataset, mean trophic positions of the 22 bird species in old‐growth forests ranged from 2.45 to 3.46. Only 15 bird species occupied the same trophic range in secondary forests, with the 6 remaining species having a trophic position above or below this range. Therefore, a wider occupied range of trophic positions and reduced interspecific competition could have resulted in non‐contracted trophic niche widths in generalists occurring in secondary forests despite forest disturbance. Another possible explanation for the missing niche width contraction in this dataset could again be provided by the high primary productivity in the studied secondary forest ecosystem, analogous to what Miller et al. (2019) documented, i.e. niche width expansion along a primary productivity gradient in coral reef fish.

Whatever the underlying reasons for the observed patterns in trophic niche widths are, non‐contracted trophic niche‐widths in bird species persisting in secondary forests highlight the already described high conservation value of this habitat (Schulze et al., 2019). Because studies showed that species with small trophic niche widths are less likely to persist after disturbance events, non‐contracted trophic niche widths render these species more resilient to future impacts compared to those persisting with contracted trophic niche widths, as documented in selectively logged forest in Borneo (Boyles & Storm, 2007; Edwards et al., 2013; Öckinger et al., 2010).

The trophic position of a species, rather than its trophic niche width, determined species persistence after disturbance in our dataset, contrary to our research hypothesis (Edwards et al., 2013). Hamer et al. (2015) have already observed that the abundance of insectivorous birds after logging is related to trophic position, with species occupying high trophic positions in old‐growth forest being most negatively affected by selective logging. Farneda et al. (2015) documented a similar effect of trophic position on the prevalence and abundance of Amazonian bats in forest fragments, again with bat species occupying high trophic positions being most negatively affected. However, Gray et al. (2007) and Newbold et al. (2013) identified frugivorous and insectivorous forest specialists as the bird species groups most susceptible to tropical forest disturbance and intensified land use, respectively. Recently, a worldwide analysis showed that small herbivorous bird species are disproportionately affected by human habitat changes (Atwood et al., 2020), and Sekercioğlu et al. (2002) pointed out that, among tropical forest birds, small understory insectivores are most sensitive to forest disturbance and fragmentation. The set of species categorised as old‐growth forest specialists in our study corresponds to these findings, as it consists of two species of frugivores (Blue‐crowned Manakin and Spot‐crowned Euphonia), four insectivorous species (Golden‐crowned Spadebill, Long‐tailed Woodcreeper, Sulphur‐rumped Flycatcher and Tawny‐crowned Greenlet) and two omnivores (Blue‐black Grosbeak and Rufous Mourner; Garrigues & Dean, 2014; Stiles & Skutch, 1989). In comparison, the set of species categorised as secondary forest specialists in this study consists of one frugivore species (White‐tipped Dove), four omnivore species (Buff‐throated Saltator, Cherrie's Tanager, Grey‐headed Tanager and Clay‐coloured Thrush) and two insectivorous species (Red‐rumped Woodpecker and Royal Flycatcher; Garrigues & Dean, 2014; Stiles & Skutch, 1989). A higher proportion of omnivores in secondary forest specialists compared to old‐growth forest specialists is in line with the documented broader trophic niche widths of secondary forest specialists.

When comparing the trophic positions of the two habitat specialist species groups, it is important to keep in mind, that no information about the main diets of these different bird species was included in the TPM. Therefore, the results may be influenced by different main diets of the investigated bird species. However, we decided not to include dietary information, such as feeding guild affiliation, in the TPM as random effect, because more detailed dietary information for these bird species was not available and trophic feeding guilds are very coarse categories. Hence, they may be imprecise and hide trophic flexibility within a guild (Edwards et al., 2013). In addition, the comparison of these two species groups is neither the main focus of this study nor is it recommended to include a random effect with only three levels (e.g. frugivorous, omnivorous and insectivorous) in such a model (Hodges, 2014).

Another factor which can explain tropical forest bird species' extinction histories and current distribution patterns is dispersal limitation (Moore et al., 2008). However, if dispersal limitations played a role in the presence–absence pattern of bird species in our dataset, it would be extremely unlikely to record such a clear pattern of trophic position determining species persistence in secondary forests after disturbance. In addition, we recorded Chestnut‐backed Antbirds in both habitat types, a species which is among the most reluctant tested to cross open‐water gaps 100 m wide (Moore et al., 2008).

## 5 CONCLUSIONS

We conclude that species feeding at low trophic levels in old‐growth forests were less likely to persist in secondary forests than those that occupied higher trophic positions in old‐growth forests. This pattern is most likely caused by relatively large‐bodied habitat specialists occurring in disturbed forests and feeding on various food sources high up the food chain. This may force co‐occurring generalist species to lower their trophic position compared to conspecifics in old‐growth forests. This probably resulted in the competitive exclusion of some species occupying low trophic positions in old‐growth forests, which were not able to lower their trophic position in secondary forests in the same way as habitat generalists occurring in secondary forests did.

To our knowledge, this is the first study to show a systematic effect of trophic position on the persistence of a wide range of bird species in a disturbed forest ecosystem. Thus, data on the trophic position of species should be incorporated into future assessments of species vulnerability to environmental changes whenever possible. In line with Edwards et al. (2013) and Hamer et al. (2015), we call for the use of flexible measures of trophic position rather than static feeding guilds, as they cannot resolve most disturbance‐induced trophic changes in a food web, such as those documented in this study. Based on our findings, we strongly recommend that ongoing conservation and reforestation efforts in the region should be intensified. Reforestation measures implemented in the past have already resulted in well‐connected secondary forests supporting numerous old‐growth forest bird species and supplying them with a broad variety of food sources. To improve habitat quality for old‐growth forest specialists not yet occurring at these sites and to facilitate avian seed dispersal of old‐growth forest plants there, forest edge and gap situations – the preferred habitat of secondary forest specialists (Garrigues & Dean, 2014; Stiles & Skutch, 1989) – should be minimised. Thus, the creation of the largest possible coherent forest patches should be prioritised when implementing reforestation measures.

## AUTHOR CONTRIBUTIONS

**Bernhard Paces:** Conceptualization (supporting); data curation (equal); formal analysis (supporting); funding acquisition (equal); investigation (lead); project administration (equal); writing – original draft (lead); writing – review and editing (equal). **Wolfgang Wanek:** Conceptualization (supporting); formal analysis (supporting); methodology (lead); writing – review and editing (equal). **Saskia Schirmer:** Formal analysis (lead); writing – review and editing (equal). **Christian C. Voigt:** Data curation (lead); methodology (lead); writing – original draft (supporting); writing – review and editing (supporting). **Paulus Leidinger:** Conceptualization (supporting); data curation (equal); investigation (lead); writing – review and editing (supporting). **Christian H. Schulze:** Conceptualization (lead); funding acquisition (equal); investigation (supporting); methodology (supporting); project administration (lead); validation (lead); writing – review and editing (lead).

## FUNDING INFORMATION

The University of Vienna supported this study financially with a Needs‐based Scholarship (Förderstipendium). There was no specific grant number provided. The German Ornithologists' Society (DO‐G) granted financial support for this research project (Forschungsförderung der DO‐G). There was no specific grant number provided. Both funding sources were not involved in any way in the design of this study, the collection, analysis and interpretation of the data, the writing of the manuscript and the decision to submit the article for publication.

## CONFLICT OF INTEREST STATEMENT

The authors declare no competing interests.

## Data Availability

All data, R Codes and further files needed to conduct the data analysis described in this manuscript can be downloaded here https://doi.org/10.6084/m9.figshare.25109213.

## APPENDIX A

**TABLE A1:** Overview of English and Latin names of the bird species from which we analysed claw material in this study.

English species name	Latin species name
Black‐cheeked Ant‐Tanager	*Habia atrimaxillaris*
Blue‐black Grosbeak	*Cyanocompsa cyanoides*
Blue‐crowned Manakin	*Lepidothrix coronata*
Bright‐rumped Attila	*Attila spadiceus*
Buff‐throated Saltator	*Saltator maximus*
Cherrie's Tanager	*Ramphocelus costaricensis*
Chestnut‐backed Antbird	*Myrmeciza exsul*
Clay‐coloured Thrush	*Turdus grayi*
Cocoa Woodcreeper	*Xiphorhynchus susurrans*
Golden‐crowned Spadebill	*Platyrinchus coronatus*
Grey‐chested Dove	*Leptotila cassini*
Grey‐headed Tanager	*Eucometis penicillata*
Long‐tailed Woodcreeper	*Deconychura longicauda*
Ochre‐bellied Flycatcher	*Mionectes oleagineus*
Orange‐billed Sparrow	*Arremon aurantiirostris*
Orange‐collared Manakin	*Manacus aurantiacus*
Red‐capped Manakin	*Ceratopipra mentalis*
Red‐rumped Woodpecker	*Veniliornis kirkii*
Riverside Wren	*Cantorchilus semibadius*
Royal Flycatcher	*Onychorhynchus coronatus*
Ruddy Quail‐Dove	*Geotrygon montana*
Rufous Mourner	*Rhytipterna holerythra*
Spot‐crowned Euphonia	*Euphonia imitans*
Sulphur‐rumped Flycatcher	*Myiobius sulphureipygius*
Tawny‐crowned Greenlet	*Hylophilus ochraceiceps*
Tawny‐winged Woodcreeper	*Dendrocincla anabatina*
Wedge‐billed Woodcreeper	*Glyphorynchus spirurus*
White‐throated Thrush	*Turdus assimilis*
White‐tipped Dove	*Leptotila verreauxi*

*Note*: Nomenclature follows Garrigues and Dean (2014).

**TABLE A2:** Model output of the trophic position model (TPM) calculated in this study.

TPM: Location effects: trophic_position ~ occurrence					
	post. Mean	l‐95% CI	u‐95% CI	eff. samp.	*pMCMC*
(Intercept)	2.9155	2.5507	3.2808	9677	< 0.0001
occurrenceOS	−0.2465	−0.4471	−0.0355	9670	0.0168
occurrenceSG	−0.2021	−0.2766	−0.1317	9998	< 0.0001
occurrenceSS	0.3665	0.1477	0.5913	9422	0.0016

	post. mean	l‐95% CI	u‐95% CI	eff. samp.
G ‐ structure: ~ species				
species	0.1472	0.0623	0.2555	9998
R ‐ structure: ~ units				
units	0.1416	0.1248	0.1585	9998

*Note*: TPM is a phylogenetically informed Markov chain Monte Carlo (MCMC) generalised linear mixed model.

Abbreviations: eff. samp., effective sample size; l‐95% CI, lower 95% credible interval; OS, old‐growth forest specialist; post., posterior; SG, secondary forest generalist; SS, secondary forest specialist; u‐95% CI, upper 95% credible interval.

**TABLE A3:** Model output of the trophic niche width model (TNWM) calculated in this study.

TNWM: Location effects: trophic_width ~ occurrence					
	post. mean	l‐95% CI	u‐95% CI	eff. samp.	*pMCMC*
(Intercept)	0.5276	0.4412	0.6060	9998	< 0.0001
occurrenceSG	−0.1772	−0.2780	−0.0746	9998	0.001
occurrenceOG	−0.2450	−0.3468	−0.1470	9998	< 0.0001
occurrenceOS	−0.2059	−0.3133	−0.0910	9689	0.0008

R ‐ structure: ~ units				
	post. mean	l‐95% CI	u‐95% CI	eff. samp.
units	0.0123	0.0070	0.0179	10,106

*Note*: TNWM is a Markov chain Monte Carlo (MCMC) generalised linear mixed model.

Abbreviations: eff. samp., effective sample size; l‐95% CI, lower 95% credible interval; OG, old‐growth forest generalist; OS, old‐growth forest specialist; post., posterior; SG, secondary forest generalist; u‐95% CI, upper 95% credible interval.

**TABLE A4:** Summary of the trophic positions calculated for fruit, moth and spider samples collected at the study sites.

SiteID	TP: Fruits	TP: Moths	TP: Spiders
CT01	1.31	2.44	2.92
FT06	0.67	3.18	2.52
LB02	1.29	2.60	2.68
OT02	0.83	2.74	3.15
SG01	0.95	2.48	2.58
LB01	1.59	2.05	2.60
LB03	1.25	2.37	3.02
VB01	0.38	2.28	2.33
VB02	0.88	3.43	3.03

Forest	M: Fruits	M: Moths	M: Spiders
OF	0.95	2.60	2.68
SF	1.06	2.34	2.81
*M‐W‐U*	10.00	5.00	10.00
*p*	.90	.27	.90

*Note*: Old‐growth forest sites (OF, *n* = 5) are separated from secondary forest sites (SF, *n* = 4) by a dashed line. Three Mann–Whitney‐*U*‐Tests were calculated to test for significant differences between forest types.

Abbreviations: M, median; *M‐W‐U*, Mann–Whitney‐*U*; TP, trophic position.

**TABLE A5:** Model output of a Gaussian Bayesian Markov chain Monte Carlo generalised linear mixed model (MCMCglmm) investigating the difference in size‐corrected body mass between forest types in habitat generalists.

MCMCglmm: Location effects: corr_mass ~ forest					
	post. mean	l‐95% CI	u‐95% CI	eff. samp.	*pMCMC*
(Intercept)	65.7730	25.7828	105.2957	9998	0.0034
forestSF	0.1486	−0.7792	1.0708	9998	0.7520

	post. mean	l‐95% CI	u‐95% CI	eff. samp.
G‐structure: ~ species				
species	1541	553	2900	9998
R‐structure: ~ units				
units	21.16	18.43	24.16	9998

Abbreviations: eff. samp., effective sample size; l‐95% CI, lower 95% credible interval; post., posterior; SF, secondary forest; u‐95% CI, upper 95% credible interval.
